# Transcriptional Profiling Reveals the Importance of RcrR in the Regulation of Multiple Sugar Transportation and Biofilm Formation in Streptococcus mutans

**DOI:** 10.1128/mSystems.00788-21

**Published:** 2021-08-24

**Authors:** Tao Gong, Xiaoya He, Jiamin Chen, Boyu Tang, Ting Zheng, Meiling Jing, Yongwang Lin, Yangyang Pan, Qizhao Ma, Yuqing Li, Xuedong Zhou

**Affiliations:** a State Key Laboratory of Oral Diseases, National Clinical Research Center for Oral Diseases, West China Hospital of Stomatology, Sichuan University, Chengdu, China; b Department of Cariology and Endodontics, West China Hospital of Stomatology, Sichuan University, Chengdu, China; c Department of Pediatric Dentistry, West China Hospital of Stomatology, Sichuan University, Chengdu, China; University of Wisconsin-Madison

**Keywords:** *Streptococcus mutans*, transcription regulator, sugar transportation, biofilm formation, dental caries

## Abstract

The ability of Streptococcus mutans to survive and cause dental caries is dependent on its ability to metabolize various carbohydrates, accompanied by extracellular polysaccharide synthesis and biofilm formation. Here, the role of an *rel* competence-related regulator (RcrR) in the regulation of multiple sugar transportation and biofilm formation is reported. The deletion of the *rcrR* gene in S. mutans caused delayed growth, decreased biofilm formation ability, and affected the expression level of its multiple sugar transportation-related genes. Transcriptional profiling revealed 17 differentially expressed genes in the *rcrR* mutant. Five were downregulated and clustered with the sugar phosphotransferase (PTS) systems (mannitol- and trehalose-specific PTS systems). The conserved sites bound by the *rcrR* promoter were then determined by electrophoretic mobility shift assays (EMSAs) and DNase I footprinting assays. Furthermore, a potential binding motif in the promoters of the two PTS operons was predicted using MEME Suite 5.1.1. RcrR could bind to the promoter regions of the two operons *in vitro*, and the sugar transporter-related genes of the two operons were upregulated in an *rcrR*-overexpressing strain. In addition, when RcrR-binding sites were deleted, the growth rates and final yield of S. mutans were significantly decreased in tryptone-vitamin (TV) medium supplemented with different sugars, but not in absolute TV medium. These results revealed that RcrR acted as a transcription activator to regulate the two PTS systems, accompanied by multiple sugar transportation and biofilm formation. Collectively, these results indicate that RcrR is a critical transcription factor in S. mutans that regulates bacterial growth, biofilm formation, and multiple sugar transportation.

**IMPORTANCE** The human oral cavity is a constantly changing environment. Tooth decay is a commonly prevalent chronic disease mainly caused by the cariogenic bacterium Streptococcus mutans. S. mutans is an oral pathogen that metabolizes various carbohydrates into extracellular polysaccharides (EPSs), biofilm, and tooth-destroying lactic acid. The host diet strongly influences the availability of multiple carbohydrates. Here, we showed that the RcrR transcription regulator plays a significant role in the regulation of biofilm formation and multiple sugar transportation. Further systematic evaluation of how RcrR regulates the transportation of various sugars and biofilm formation was also conducted. Notably, this study decrypts the physiological functions of RcrR as a potential target for the better prevention of dental caries.

## INTRODUCTION

Dental caries is a prevalent and consequential oral disease worldwide (1). In 2016, caries of permanent teeth was among the top 10 causes of the most pervasive disorders (2). Tooth decay arises from biofilms containing cariogenic microorganisms that break down fermentable carbohydrates into organic acids. These acids subsequently cause demineralization and destruction of the tooth tissue (3). Streptococcus mutans is a primary cariogenic pathogen that utilizes glucosyltransferases (GtfB, GtfC, and GtfD) to synthesize extracellular polysaccharides (EPSs) from sucrose (4), thereby providing adhesive sites for the colonization of other microorganisms. This occurrence leads to the formation of cariogenic biofilms (5). Keeping this in mind, EPS synthesis and biofilm formation by S. mutans are crucial virulence factors in the pathogenesis of dental caries.

Sugar transportation and metabolism play significant roles, such as EPS synthesis, biofilm formation, and lactic acid production, in the physiology and virulence of S. mutans (6). There are two primary sugar incorporation systems reported in S. mutans, phosphotransferase (PTS) systems and non-PTS systems (6). These systems are responsible for binding, transmembrane transportation, and phosphorylation of various sugar substrates. Meanwhile, they also regulate various metabolic and transcriptional processes and virulence factors, such as growth, energy metabolism, competence, EPS production, and biofilm formation (6–10). An increasing number of studies have shown that genes coding for sugar transportation and metabolism in S. mutans are regulated by transcription factors, sigma factors, and two-component regulatory systems (11, 12). Recently, some transcription factors involved in sugar metabolism have been most studied in S. mutans. For example, TreR, CelR, and StsR act as transcriptional activators that regulate the trehalose operon, cellobiose operon, and multiple transporter genes, respectively (12–14). However, there are also negative regulators. For instance, FruR and NigR negatively regulate the transcription of the *fruRKI* operon and the PTS^Bio^ operon, respectively (9, 15).

Here, a new function of *rel* competence-related regulator (RcrR; *SMU_921*) was discovered. RcrR is a multiple antibiotic resistance regulator (MarR) family transcription factor in S. mutans UA159. Previously, RcrR has been demonstrated to play critical roles in environmental stresses, genetic competence, and (p)ppGpp metabolism in S. mutans (16). In this study, the loss of *rcrR* influenced bacterial cell growth, multiple sugar transportation, and biofilm formation. Further investigations via mRNA sequencing of an *rcrR* mutant revealed that five genes in two PTS systems (mannitol- and trehalose-specific PTS systems) that were involved in sugar transportation were significantly downregulated. Thus, our findings indicate that RcrR is the transcription activator of two PTS systems that control multiple sugar utilization and biofilm formation.

## RESULTS

### Deletion of *rcrR* causes growth delay in S. mutans.

**FIG 1:**
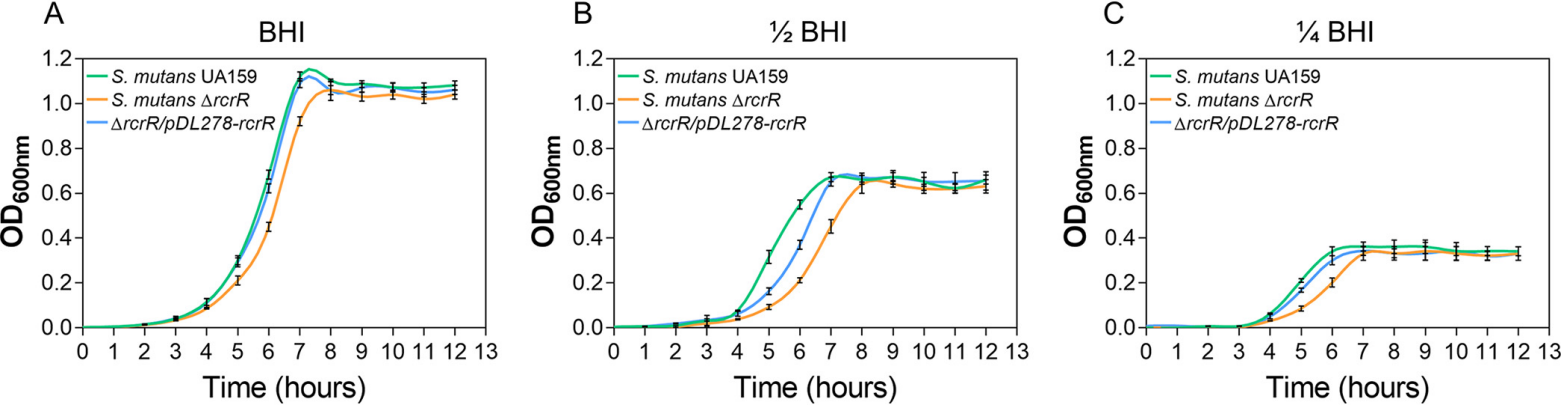
Effect of *rcrR* deletion on bacterial growth in Streptococcus mutans. Growth curves of S. mutans UA159, S. mutans Δ*rcrR*, and S. mutans Δ*rcrR*/*pDL278*-*rcrR* strains in brain heart infusion (BHI) (A), 1/2 BHI (B), and 1/4 BHI (C) media for 12 h under anaerobic conditions. See Table S2 in the supplemental material for data regarding the analysis of significance among these curves.

*SMU_921* (*rcrR*) can be cotranscribed with *SMU_922* (*rcrP*) and *SMU_923* (*rcrQ*). As such, they are annotated as transmembrane ATP-binding cassette (ABC) transporters in a polycistronic operon (16). Studies have previously focused on RcrR regulation of *rcrP* and *rcrQ* expression and physiological functions (16). Here, we found that RcrR possesses other important functions. Growth delay of the *rcrR* mutant (Δ*rcrR*) was observed regardless of the nutritional condition (brain heart infusion [BHI], 1/2 BHI, and 1/4 BHI media) (Fig. 1; *P < *0.05). Meanwhile, the Δ*rcrR* mutant showed an extended lag phase in the 1/2 BHI medium compared with those of the wild-type and complement strains (Fig. 1B). Nonetheless, the cells of all strains reached the same optical density at the stationary phase. These results indicated that *rcrR* deficiency affected the growth of planktonic bacteria.

10.1128/mSystems.00788-21.3 TABLE S2 Analysis of growth curve significance of different strains cultured in one specific medium. Download Table S2, XLSX file, 0.01 MB. Copyright © 2021 Gong et al. 2021 Gong et al. https://creativecommons.org/licenses/by/4.0/ This content is distributed under the terms of the Creative Commons Attribution 4.0 International license.

### Effect of *rcrR* deficiency on the growth of S. mutans with different sugar supplementations.

**FIG 2:**
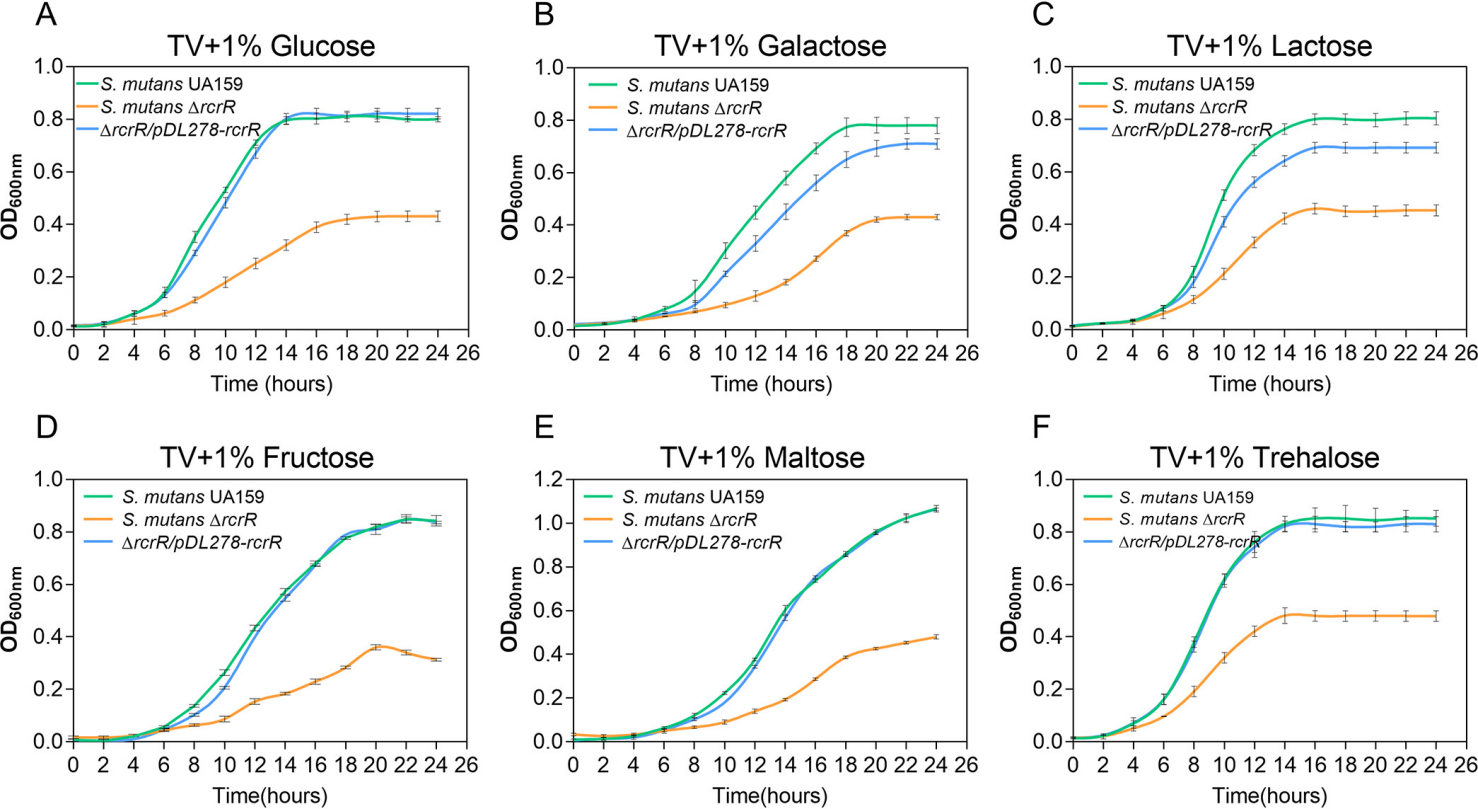
Effect of *rcrR* deficiency on the growth of S. mutans with different sugar supplementations. Growth curves of S. mutans UA159, S. mutans Δ*rcrR*, and S. mutans Δ*rcrR*/*pDL278*-*rcrR* strains in tryptone-vitamin (TV) medium supplemented with glucose (A), galactose (B), lactose (C), fructose (D), maltose (E), and trehalose (F) (1%, wt/vol) for 24 h under anaerobic conditions. See Table S2 for data regarding the analysis of significance among these curves.

Based on the findings of growth delays caused by *rcrR* deficiency in different nutritional conditions (Fig. 1), it was intriguing to speculate on a specific relationship between *rcrR* and multiple sugar utilization. The growth curves of the wild-type, the Δ*rcrR*, and the complement strains were thus measured when incubated in TV medium supplemented with common sugars, including glucose, galactose, lactose, fructose, maltose, and trehalose (1% [wt/vol]), to verify this phenomenon (12, 17). All Δ*rcrR* mutants had a significantly extended lag phase and decreased final yield compared with those of the wild-type and the complement strains (Fig. 2; *P < *0.05). The different growth rates and final yield showed that the ability to utilize diverse sugars was also dramatically different between S. mutans strains. The growth rate and final yield of complement strains incubated in TV medium plus galactose/lactose also gradually decreased (Fig. 2B and C). These results also illustrated that *rcrR* deficiency had an important effect on the bacterial growth of S. mutans with different sugar supplementations. S. mutans does not grow in TV medium without carbohydrate supplementation (18). It is possible that *rcrR* deletion inhibited the transportation or metabolism of multiple sugars of S. mutans, indirectly affecting bacterial growth.

### Biofilm formation of S. mutans is susceptible to *rcrR* deletion.

**FIG 3:**
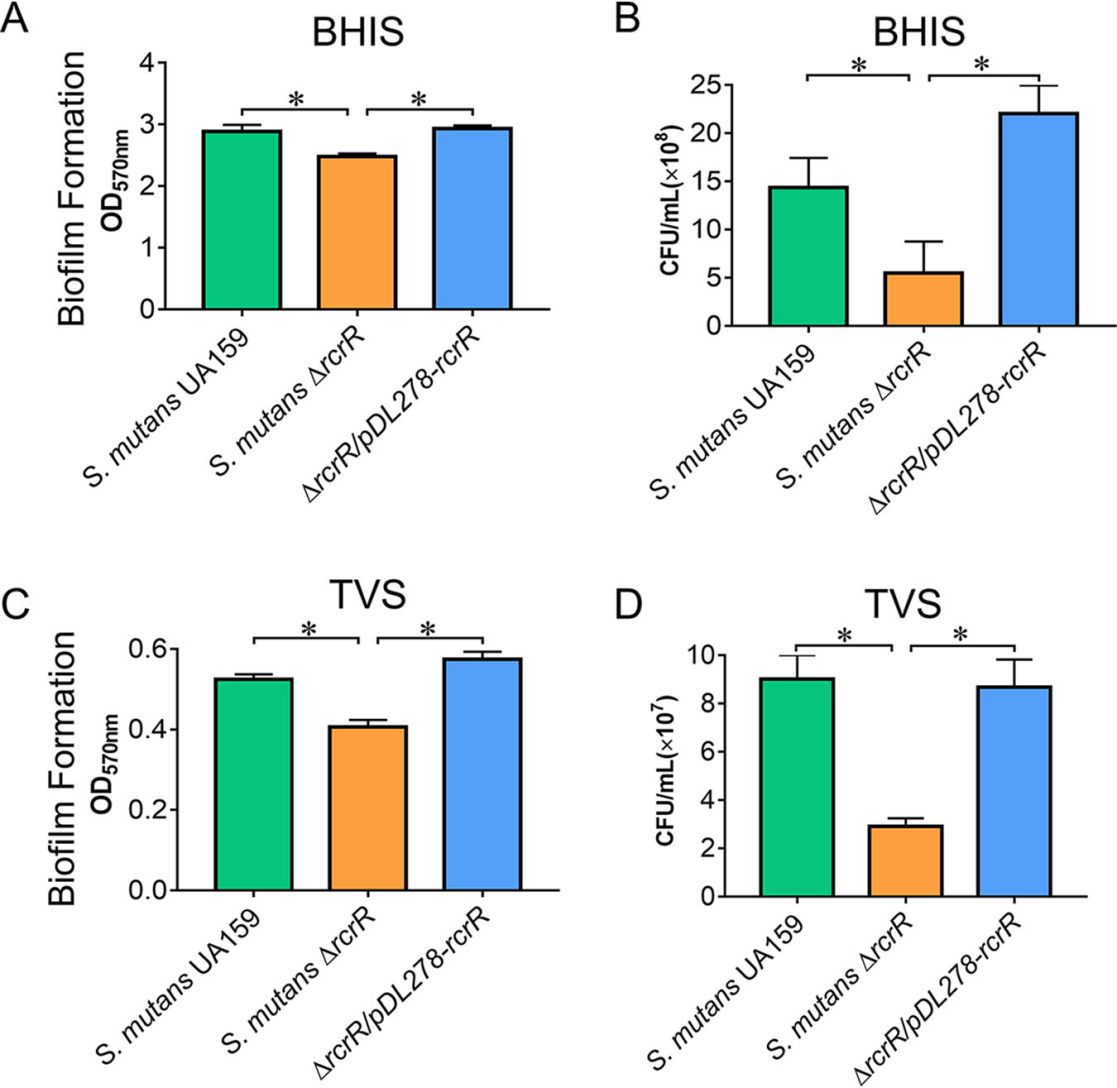
Biofilm formation of S. mutans is susceptible to the deletion of *rcrR*. The total biofilm biomass was determined by crystal violet staining when S. mutans was cultured in BHIS (BHI plus 1% sucrose [wt/vol]) (A) and TVS (TV plus 1% sucrose) (C) media for 24 h under anaerobic conditions. CFU from the biofilms were counted (B and D). Data are presented as means ± standard deviation (SD) (*n* = 4; *, *P < *0.05).

The ability to form a sucrose-dependent biofilm is regarded as one of the most important virulence factors in S. mutans. Biofilms of the wild-type, the Δ*rcrR*, and the complement strains were further quantified by crystal violet staining assays (Fig. 3A and C). The Δ*rcrR* biofilms were significantly reduced compared to the biofilms of the wild-type and complement strains when cultured in BHI plus 1% sucrose (BHIS) or TV plus 1% sucrose (TVS) medium (Fig. 3A and C; *P < *0.05). The total CFU from biofilms were then counted, and we found that the CFU from the Δ*rcrR* mutant were also decreased compared to those from the wild-type and complement strains (Fig. 3B and D; *P < *0.05). These results demonstrated that the deletion of the *rcrR* gene led to the reduction of bacterial growth and biofilm formation, suggesting the decline in biofilm formation ability was due to a decrease in the number of bacteria.

**FIG 4:**
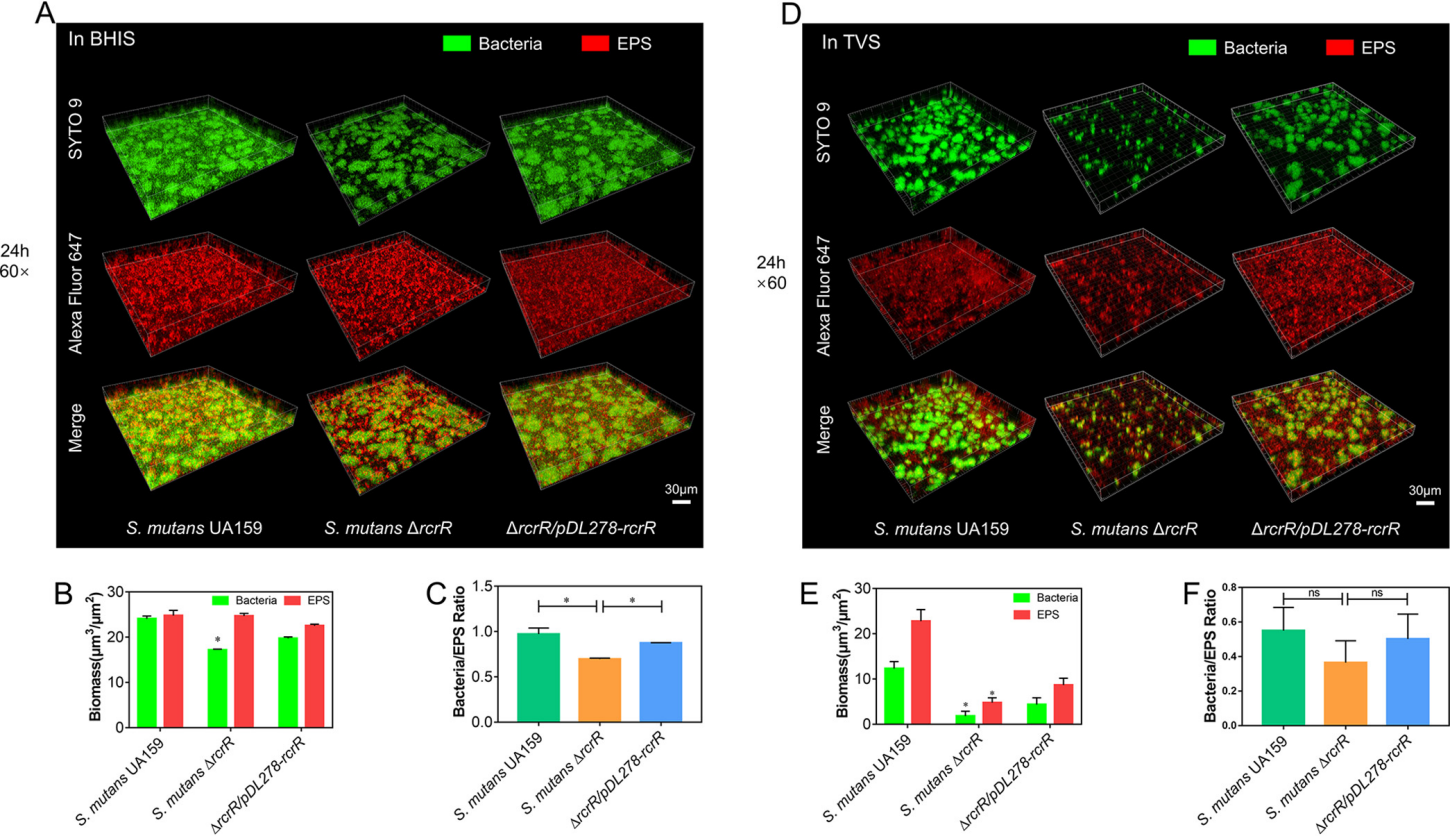
Biofilm formation of S. mutans is susceptible to the deletion of *rcrR*. Double labeling of S. mutans biofilms formed in BHIS (A) and TVS (D) media. Green denotes bacteria (SYTO 9), and red denotes exopolysaccharide (EPS) (Alexa Fluor 647). Confocal laser scanning microscopy (CLSM)-captured images (×60 magnification). Three-dimensional reconstruction of the biofilms was performed with Imaris 9.0.1. Representative images were displayed from at least three randomly selected positions in each sample. Quantification of bacteria and EPS biomass (B and E) was conducted, and the ratio of bacteria to EPS (C and F) was calculated using Comstat 2.1. Results are the average of three randomly selected positions in each sample. Data are presented as mean ± SD (*, *P < *0.05).

The 24-h time point was selected to visualize the biofilm using a confocal laser scanning microscope (CLSM) after culturing in BHIS or TVS medium (Fig. 4A or D). The representative three-dimensional images of bacteria (green) and EPS (red) in S. mutans biofilms are shown in Fig. 4. In the BHIS medium, the Δ*rcrR* biofilm contained fewer bacteria (Fig. 4A, upper, green) than those observed in wild-type and complement strain biofilms. However, EPS (Fig. 4A, middle, red) showed no apparent differences, which is consistent with fluorescence quantitation of total bacterial and EPS biomass (Fig. 4B; *P < *0.05). Meanwhile, the ratio of bacteria to EPS in Δ*rcrR* biofilm was significantly lower than that in wild-type and complement strain biofilms (Fig. 4C; *P* < 0.05). In the TVS medium, the biofilm formed by the Δ*rcrR* mutant contained fewer bacteria (Fig. 4D, upper, green) and less EPS matrix (Fig. 4D, middle, red) than those of wild-type and complement strains, similar to the quantitative analysis of total bacteria and EPS biomass (Fig. 4E; *P* < 0.05). However, there were no significant differences in the ratio of bacteria to EPS among these strains (Fig. 4F). In line with the results shown in Fig. 3, these data also indicated that bacterial growth (CFU) and biofilm formation of S. mutans were susceptible to the deletion of *rcrR*, regardless of the nutritional conditions (BHIS or TVS).

### Transcriptomic analysis of the S. mutans Δ*rcrR* mutant.

**FIG 5:**
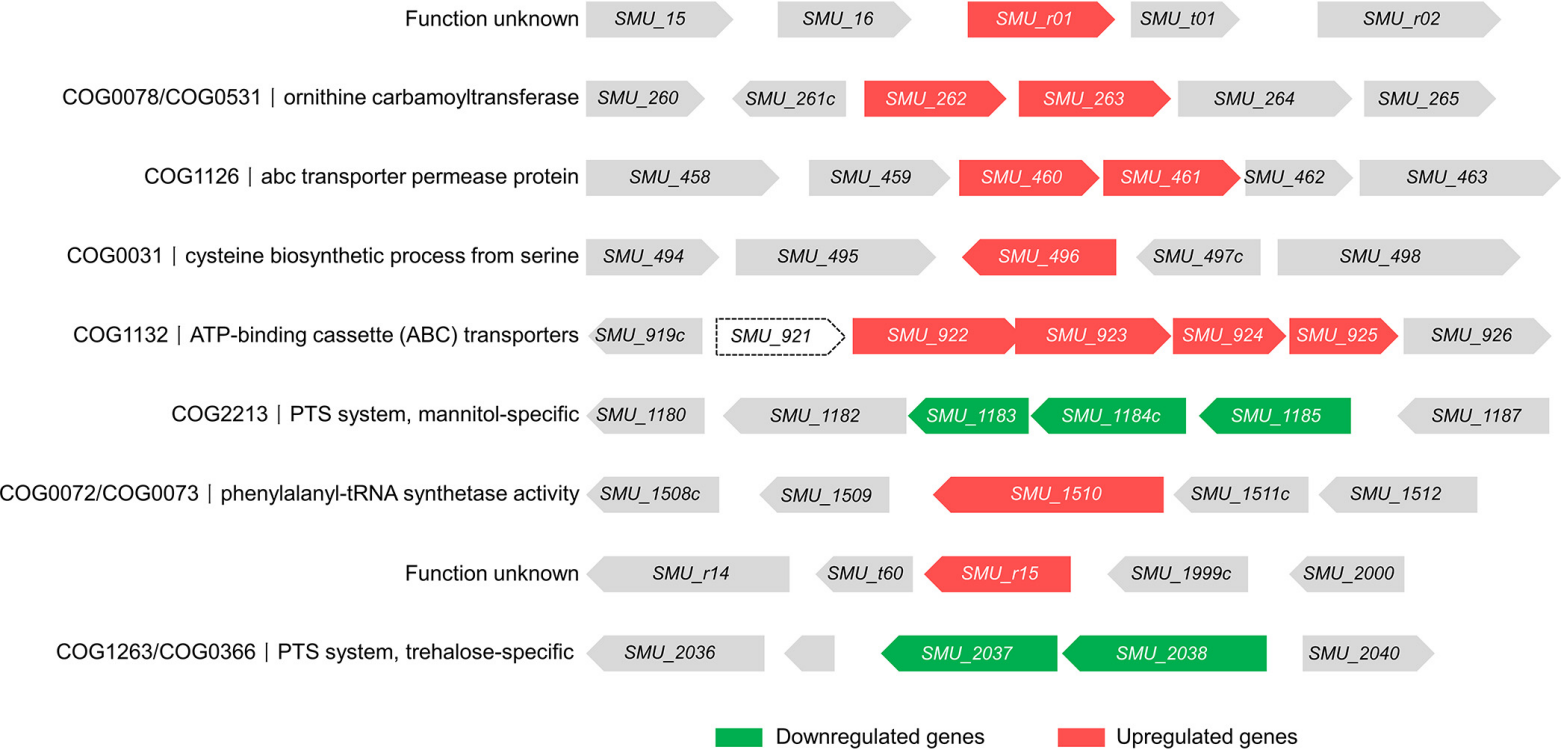
Genetic organization of all differentially expressed gene clusters and clusters of orthologous groups (COG) function description in the S. mutans Δ*rcrR* strain. The dashed-line box denotes gene deletion. Upregulated and downregulated genes are colored red and green, respectively.

**FIG 6:**
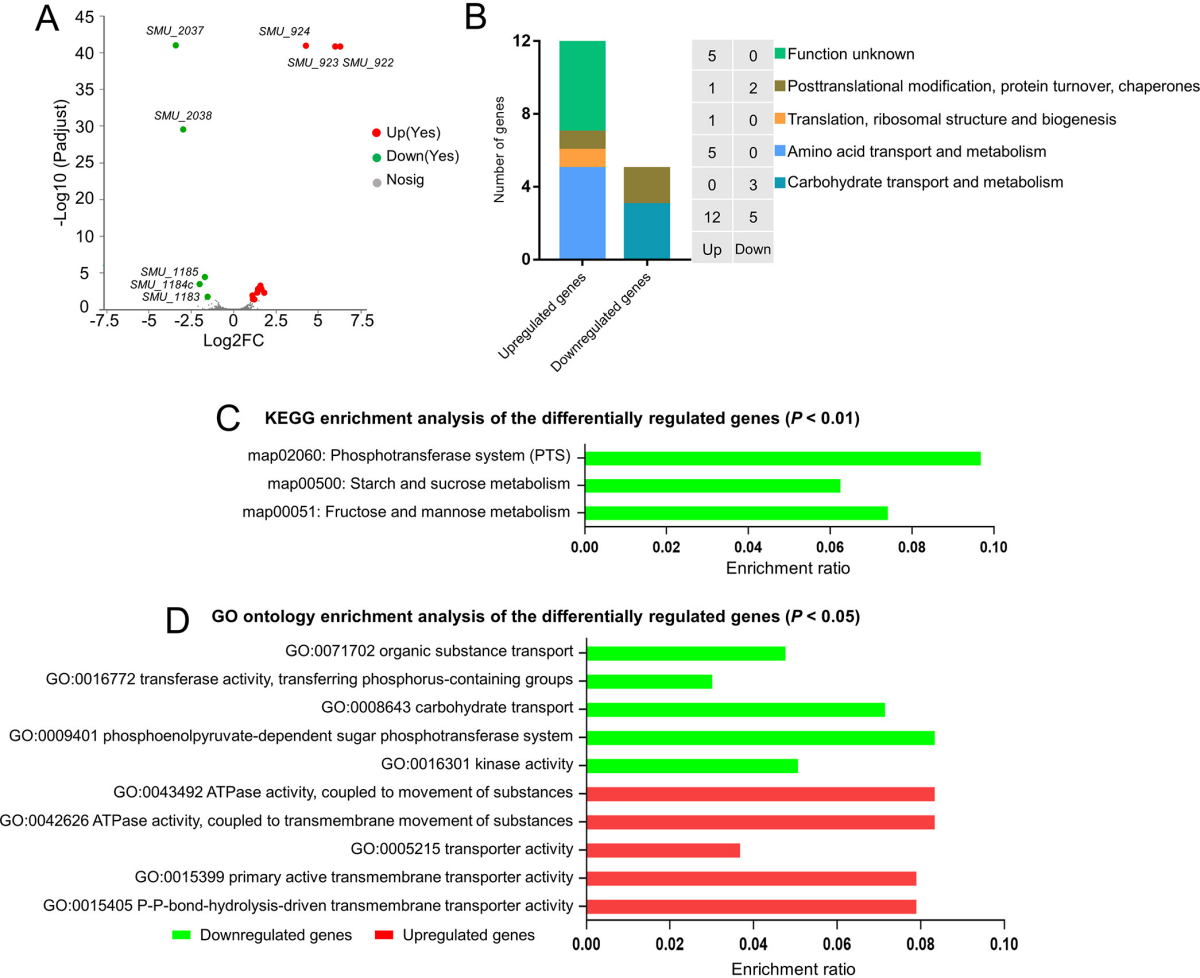
Transcriptomic analysis of the S. mutans Δ*rcrR* mutant. (A) A volcano plot revealing the differences in gene expression between the Δ*rcrR* strain and the wild type. (B) Classification and percentage of the differently expressed genes (DEGs) were performed based on the COG type. (C) Functional categories and Kyoto Encyclopedia of Genes and Genomes (KEGG) enrichment analysis of DEGs using the Majorbio cloud platform (*P* < 0.01). (D) Functional categories and gene Ontology (GO) enrichment analysis of DEGs using the Majorbio cloud platform (73 terms were identified; the first 5 enrichment terms of upregulated and downregulated genes are shown based on the *P* values from low to high, respectively; *P* < 0.05). Red and green denote the upregulated and downregulated genes, respectively.

Transcriptome analysis was performed to examine the gene expression in the *rcrR* mutant strain. There were 17 differentially expressed genes (DEGs) between UA159 and the *rcrR* mutant (Fig. 5; see also Table S3 in the supplemental material; *P < *0.05). Of these, 12 genes were significantly upregulated, and 5 genes were downregulated in the *rcrR* mutant compared to the wide-type strain (Fig. 5 and Fig. 6A and B). These genes were mainly associated with posttranslational modifications, protein turnover, chaperones, translation, ribosomal structure, and biogenesis (Fig. 6B). They were also associated with amino acid transport and metabolism and carbohydrate transport and metabolism (Fig. 6B). The DEGs were found to be enriched in three Kyoto Encyclopedia of Genes and Genomes (KEGG) pathways and 73 gene ontology (GO) terms by bioinformatics analyses (Majorbio Co., Ltd., China) (Fig. 6C and D and Tables S4 to S7). Notably, the downregulated genes during KEGG pathway analysis included those involved in the PTS system, starch and sucrose metabolism, and fructose and mannose metabolism (Fig. 6C; *P* < 0.05). Similarly, they primarily belonged to various transport systems (sorting *P* values corrected from low to high), including kinase activity and the phosphoenolpyruvate-dependent sugar phosphotransferase system (Fig. 6D). They were also associated with carbohydrate transport, transferase activity, and organic substance transport (Fig. 6D and Table S6; *P < *0.05). These findings revealed that the deletion of *rcrR* downregulated the sugar transportation-related genes, leading to a decrease in transportation of multiple sugars, further influencing the bacterial growth and biofilm formation of S. mutans.

10.1128/mSystems.00788-21.4 TABLE S3 All differentially expressed genes in the S. mutans Δ*rcrR* strain (*P < *0.05). Download Table S3, XLSX file, 0.01 MB. Copyright © 2021 Gong et al. 2021 Gong et al. https://creativecommons.org/licenses/by/4.0/ This content is distributed under the terms of the Creative Commons Attribution 4.0 International license.

10.1128/mSystems.00788-21.5 TABLE S4 Kyoto Encyclopedia of Genes and Genomes (KEGG) enrichment analysis of the downregulated genes in the S. mutans Δ*rcrR* strain (*P < *0.01). Download Table S4, XLSX file, 0.01 MB. Copyright © 2021 Gong et al. 2021 Gong et al. https://creativecommons.org/licenses/by/4.0/ This content is distributed under the terms of the Creative Commons Attribution 4.0 International license.

10.1128/mSystems.00788-21.6 TABLE S5 KEGG enrichment analysis of the upregulated genes in the S. mutans Δ*rcrR* strain (*P*>0.05). Download Table S5, XLSX file, 0.01 MB. Copyright © 2021 Gong et al. 2021 Gong et al. https://creativecommons.org/licenses/by/4.0/ This content is distributed under the terms of the Creative Commons Attribution 4.0 International license.

10.1128/mSystems.00788-21.7 TABLE S6 Gene ontology (GO) enrichment analysis of the downregulated genes in the S. mutans Δ*rcrR* strain (*P < *0.05). Download Table S6, XLSX file, 0.01 MB. Copyright © 2021 Gong et al. 2021 Gong et al. https://creativecommons.org/licenses/by/4.0/ This content is distributed under the terms of the Creative Commons Attribution 4.0 International license.

### RcrR directly binds to its own operon promoter.

**FIG 7:**
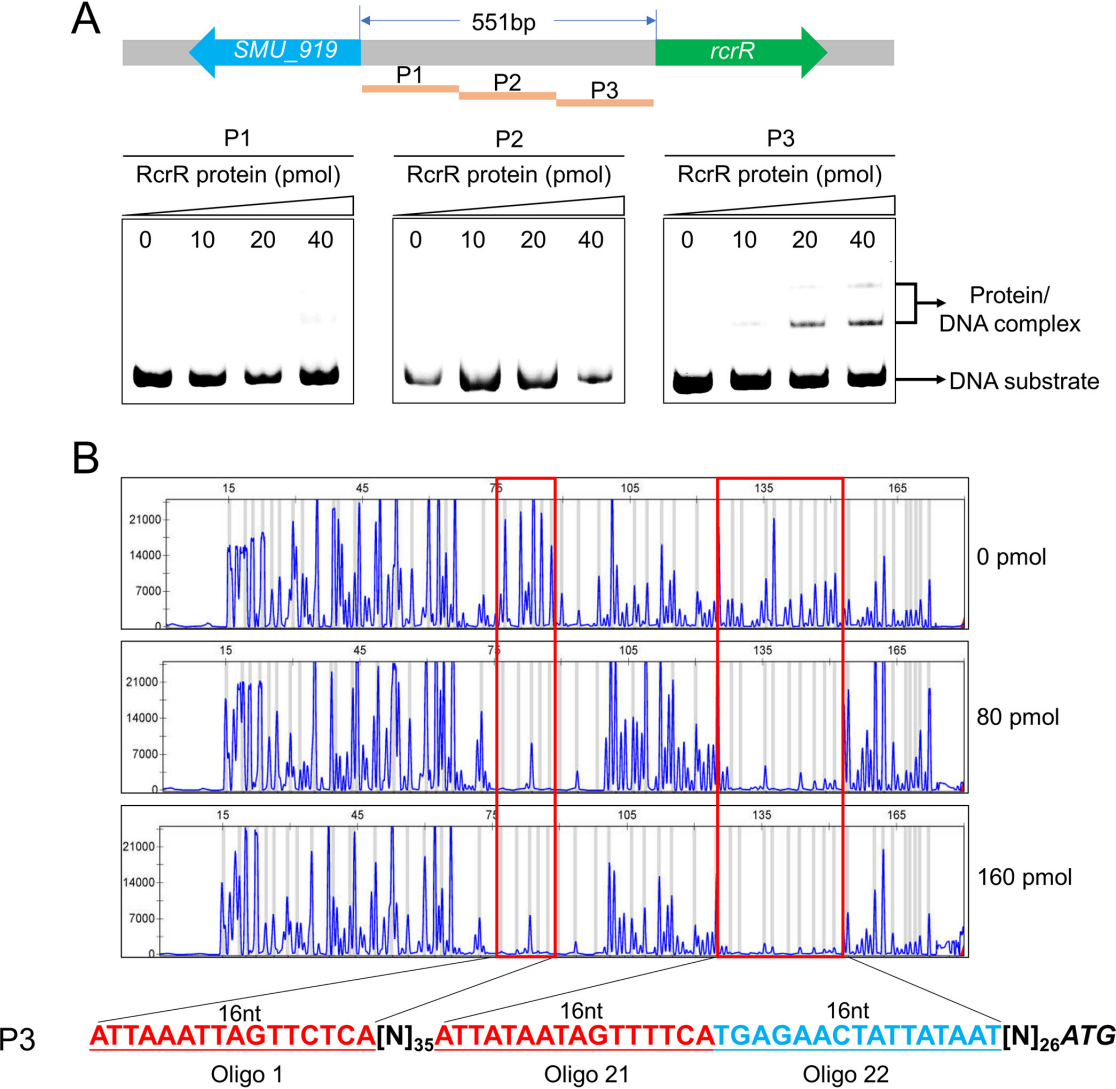
Identification of the conserved DNA motif bound by RcrR. (A) Interaction of RcrR protein with its promoter. The *rcrR* promoter was divided into three DNA fragments, P1, P2, and P3. Each fragment (20 pmol) was incubated with different amounts of RcrR. Electrophoretic mobility shift assay (EMSA) results showing RcrR binding to these DNA substrates. (B) DNase I footprinting assays for the identification of DNA motif bound by RcrR. Protection of RcrR promoter against DNase I digestion by increasing the amount of RcrR. The protected regions of the sequences are marked in the figure (Oligo 1, oligonucleotide 1; Oligo 21, oligonucleotide 21; Oligo 22, oligonucleotide 22). The ATG start codon of the *rcrR* gene is highlighted and in italics.

The 551-bp spacer region between *SMU_919* and *SMU_921* (*rcrR*) was divided into three fragments of equal lengths (P1 and P2 = 184 bp each; P3 = 183 bp) to study the DNA binding activity and specificity of RcrR. The three DNA fragments incubated with purified recombinant His-RcrR protein were then subjected to an electrophoretic mobility shift assay (EMSA). There were mobility shifts when RcrR was incubated with the P3 fragment. However, there were no shifts when it was incubated with the P1 and P2 fragments (Fig. 7A). When the P3 fragment-RcrR protein complex was exposed to DNase I, three regions, namely, 5′-ATTAAATTAGTTCTCA-3′ (oligonucleotide 1), 5′-ATTATAATAGTTTTCA-3′ (oligonucleotide 21), and 5′-TGAGAACTATTATAAT-3′ (oligonucleotide 22), were protected by RcrR (Fig. 7B). Interestingly, oligonucleotide 21 plus oligonucleotide 22 was a palindromic sequence with high AT content. These results indicated that RcrR is specifically bound to its promoter region.

### Positive regulation of two PTS systems by RcrR.

**FIG 8:**
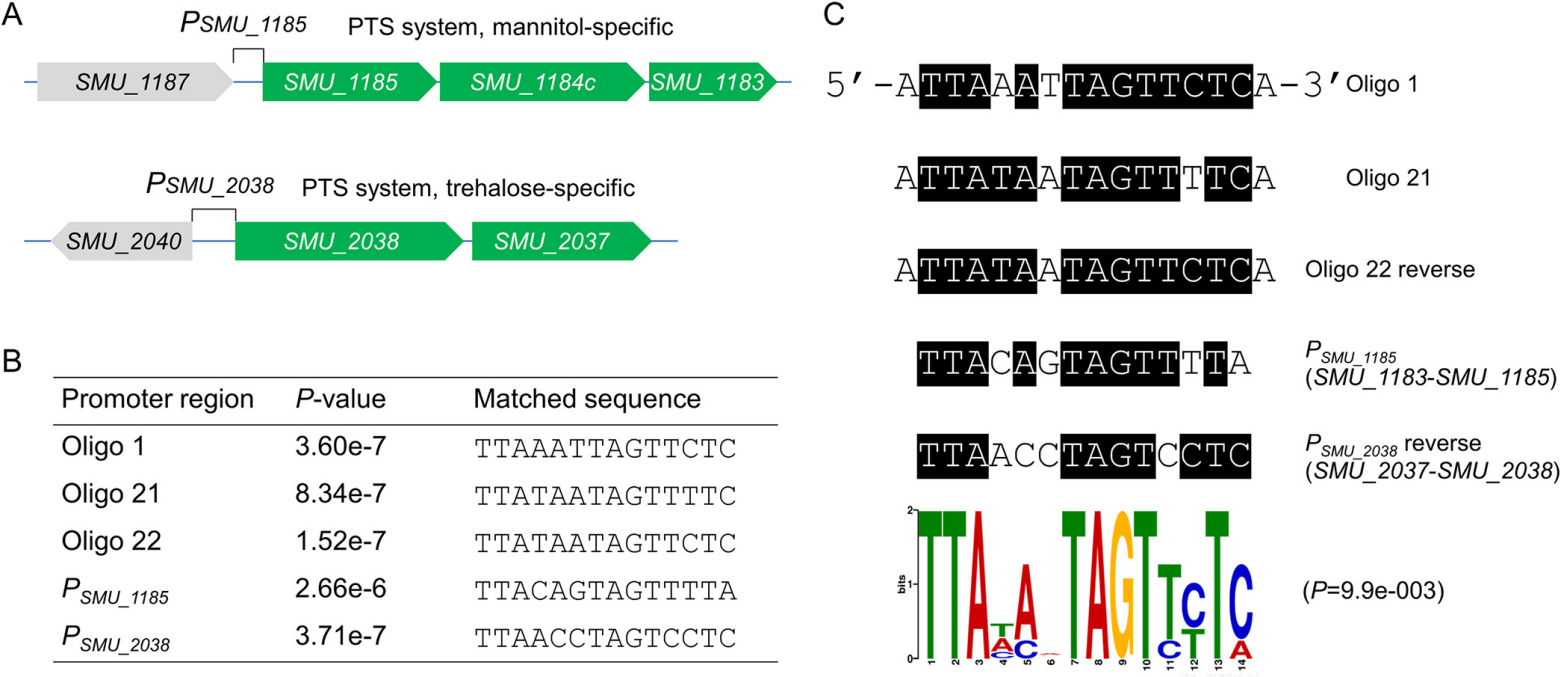
Predicting the potential binding sites of RcrR. (A) The operon promoters of mannitol-specific (*P_SMU_1185_*) and trehalose-specific (*P_SMU_2038_*) phosphotransferase (PTS) systems were used to search for a generally conserved motif (B) for RcrR binding using MEME Suite 5.1.1. (C) A predicted sequence logo for the RcrR binding motif was generated using MEME Suite 5.1.1 (*P < *0.05).

**FIG 9:**
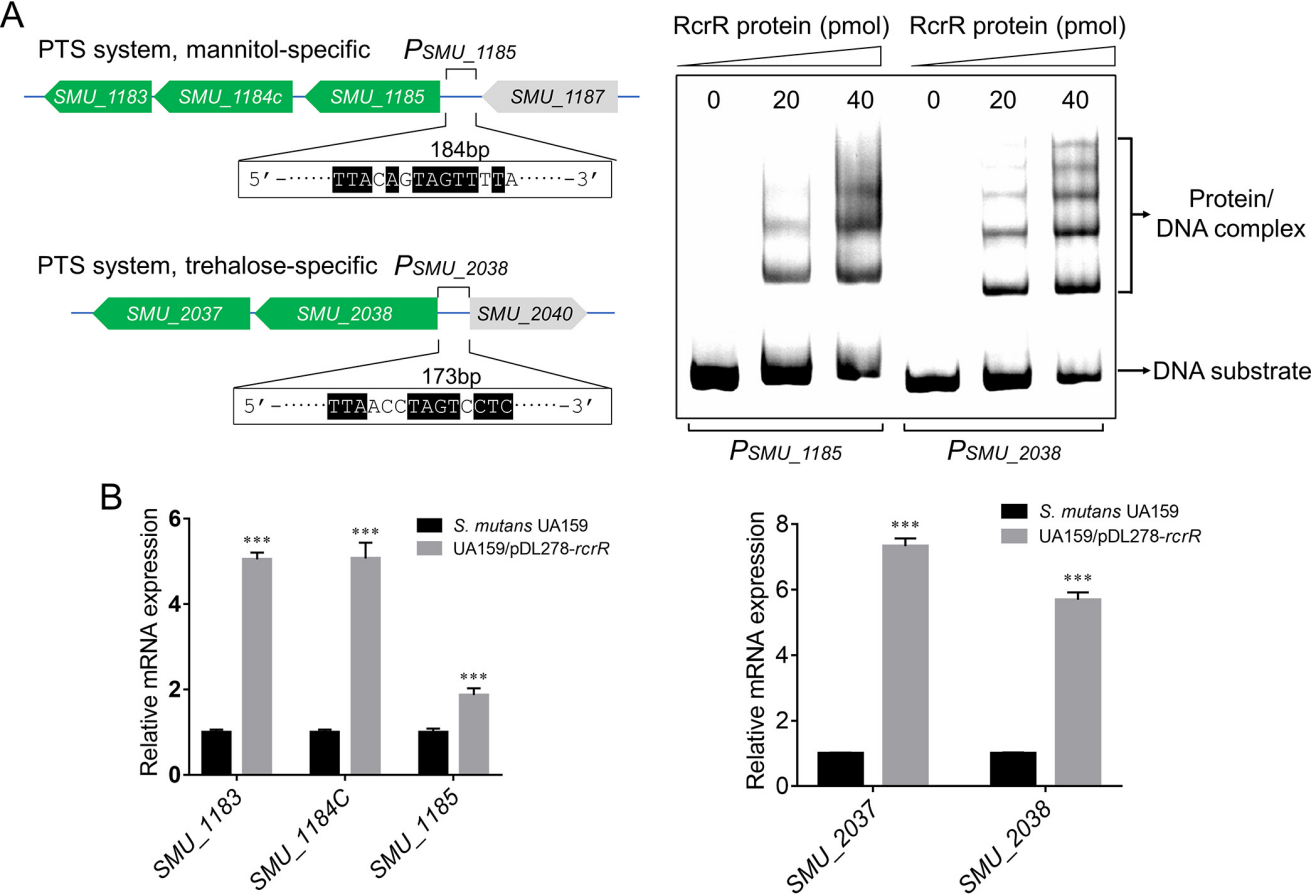
Positive regulation of two PTS systems by RcrR. (A) Interaction of RcrR protein with the promoter regions of the two PTS operons, *P_SMU_1185_* and *P_SMU_2038_*. Each fragment (20 pmol) was incubated with different amounts of RcrR. EMSA results showing RcrR binding to these DNA substrates. (B) Comparison of the expression levels of two operon genes in the wild-type and *rcrR*-overexpressing strains by quantitative reverse transcription-PCR (qRT-PCR). Data are presented as mean ± SD from three independent experiments (*n* = 4; ***, *P < *0.001).

There were only 5 downregulated genes based on transcriptome sequencing (RNA-seq) results (Fig. 5), which were classified by two sugar transporter operons, the mannitol-specific PTS system (*SMU_1183*, *SMU_1184c*, and *SMU_1185*) and the trehalose-specific PTS system (*SMU_2037* and *SMU_2038*) (Fig. 8A). The promoter regions (*P_SMU_1185_* and *P_SMU_2038_*) were used to search for a generally conserved motif for RcrR binding using MEME Suite 5.1.1 (Fig. 8B), generating a 14-nucleotide (nt) sequence logo (Fig. 8C; *P* < 0.05). The deletion of *rcrR* induced the downregulation of two PTS operon genes, suggesting that RcrR can function as an activator to regulate the expression of downstream genes. An *rcrR*-overexpressing UA159 strain, containing plasmid *pDL278*-*rcrR*, was constructed and was used to verify whether RcrR could upregulate two PTS operons. The expression levels of *SMU_1183*, *SMU_1184c*, *SMU_1185*, *SMU_2037*, and *SMU_2038* in the *rcrR*-overexpressing strain were found to be upregulated 2- to 7-fold compared with the wild-type strain by quantitative reverse transcription-PCR (qRT-PCR) (Fig. 9B). The predicted binding sequences were then subjected to EMSA with RcrR to further investigate this phenomenon *in vitro*. The mobility shift result generated many DNA-protein complex strips and showed that RcrR could more specifically bind to *P_SMU_1185_* and *P_SMU_2038_* compared with the corresponding controls of P1 and P2 (Fig. 9A).

**FIG 10:**
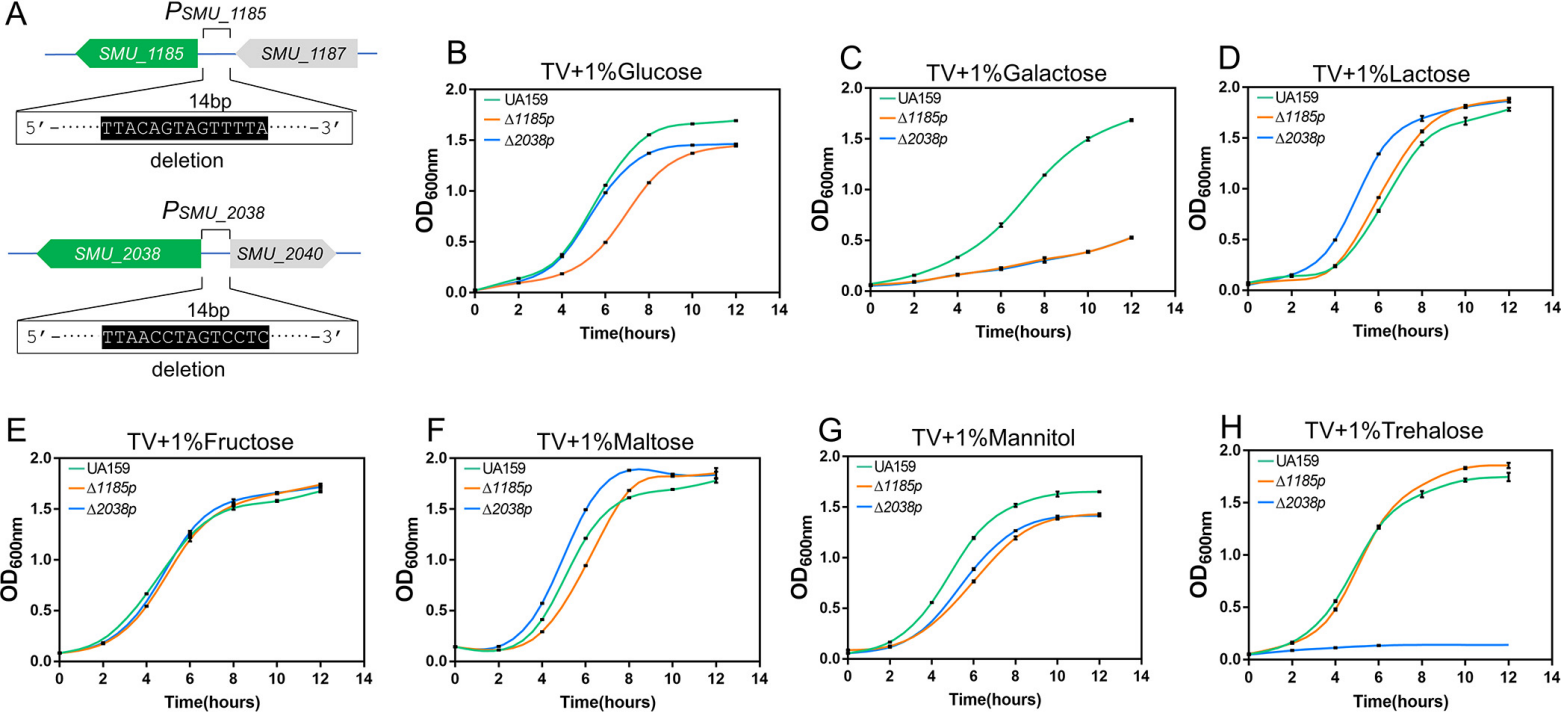
RcrR binding to *P_SMU_1185_* and *P_SMU_2038_* may positively regulate multiple sugar transportation of S. mutans. (A) Schematic diagram of Δ*1185p* and Δ*2038p* construction mutants with the deletion of binding sites of RcrR protein in the *P_SMU_1185_* and *P_SMU_2038_* promoter region. (B to H) Growth curves of UA159, Δ*1185p*, and Δ*2038p* strains in TV medium supplemented with glucose (B), galactose (C), lactose (D), fructose (E), maltose (F), mannitol (G), and trehalose (H) (1%, wt/vol) for 12 h under anaerobic conditions. See Table S2 for data regarding the analysis of the significance of these curves.

To further verify whether RcrR binds with *P_SMU_1185_* and *P_SMU_2038_* to activate the expression of the two PTS operons responsible for transporting multiple sugars *in vivo*, we constructed two mutants (Δ*1185p* and Δ*2038p*) in which the RcrR binding sites were deleted (Fig. 10A). The growth curves of UA159, Δ*1185p*, and Δ*2038p* were then measured in the presence of different sugars (Fig. 10B to H). We found that the growth rates and final yield of Δ*1185p* and Δ*2038p* decreased compared to the those of the wild-type strain when cultured in TV plus glucose/galactose/mannitol medium (Fig. 10B, C, and G). Simultaneously, the growth rate and final yield of Δ*2038p* significantly decreased compared to those of the wild-type and Δ*1185p* strains when cultured in TV plus trehalose medium (Fig. 10H). However, the growth rates and final yield of the three strains showed unstable levels when cultured in TV plus lactose/fructose/maltose medium (Fig. 10D to F). These results demonstrated that the deletion of RcrR binding sites decreased the capacity for multiple sugar transportation and the bacterial growth of S. mutans, but not completely. In brief, RcrR binding with the predicted sites was likely to function as a transcription activator to positively regulate the expression of the two PTS operons in S. mutans.

## DISCUSSION

Biofilm formation of S. mutans plays an extremely important role in the development of dental caries. Sugar transport and metabolism participate in the regulation of EPS synthesis and biofilm formation, via the PTS systems and ABC transporters (6, 11). The PTS system can also be regulated by various regulatory factors (11, 12). Here, we found that the expression of the two PTS systems was susceptible to *rcrR* deletion. This phenomenon resulted in decreased growth, biofilm formation, and multiple sugar transportation. This study provides further insights into the mechanisms of RcrR as a transcription activator that manipulates two PTS systems by directly binding to their promoter regions.

Previous studies have found that the *rcrRPQ* operon plays a critical role in physiological homeostasis and stress tolerance by linking acid and oxidative stress tolerance, (p)ppGpp metabolism, and genetic competence in S. mutans (16). It is well established in the literature that different mutant constructs of the *rcrR* gene (including Δ*rcrR*-P for a polar mutation and Δ*rcrR*-NP for a nonpolar mutation) can cause different gene expression changes (16, 19). Our method for constructing an Δ*rcrR* mutant is based on the markerless mutagenesis protocol using the IFDC2 cassette that absolutely deletes the open reading frame (ORF) (from ATG to TAA) (20). Interestingly, there are extreme differences in gene expression profiling between the RNA-seq results of Kaspar et al. for Δ*rcrR*-P and Δ*rcrR*-NP strains (19) and our RNA-seq results for the Δ*rcrR* strain. Only the expression levels of a few genes were consistent. For example, the gene expression levels of the *rcrRPQ* operon and *SMU_1185* were upregulated and downregulated in both strains (Δ*rcrR*-NP and our Δ*rcrR* mutants). We further verified the expression levels of *rcrRPQ* operon in the wild-type, mutant, complement, and *rcrR*-overexpressing strains by qRT-PCR (see Fig. S1 in the supplemental material), consistent with our RNA-seq results. The above-mentioned results suggested that the phenotype of our mutant was partially, but not absolutely, similar to that of the Δ*rcrR*-NP mutant. It is possible that S. mutans has developed various strategies (including changing the expression levels of different genes) in response to different *rcrR* mutations caused by complex environmental stresses in the oral cavity.

10.1128/mSystems.00788-21.1 FIG S1 The expression level of the *rcrRPQ* operon was determined by quantitative reverse transcription-PCR (qRT-PCR) in different strains. 16S rRNA was used as an internal control. Data are presented as means ± standard deviation (SD) of three independent experiments. Download FIG S1, TIF file, 1.1 MB. Copyright © 2021 Gong et al. 2021 Gong et al. https://creativecommons.org/licenses/by/4.0/ This content is distributed under the terms of the Creative Commons Attribution 4.0 International license.

One more difference between this RNA-seq study of an *rcrR* mutant and the Kaspar et al. RNA-seq study is that our study was completed in an anaerobic environment and that of Kaspar et al. was completed in 5% CO_2_ atmosphere (oxygen) (19). A previous study demonstrated that the biochemical and phenotypic properties of S. mutans were changed during growth with aeration compared with growth under an anaerobic condition (21, 22). In particular, S. mutans bacteria that initially colonize an oral surface are exposed to higher levels of oxygen than those in mature biofilms, where diffusion of oxygen appears to be limited (22). Thus, S. mutans may regulate the expression level of the corresponding genes in response to exposure to oxygen. Seaton et al. showed that Δ*rcrP*-NP and Δ*rcrQ*-NP mutants were sensitive to oxidative stressors (16), suggesting that the *rcrRPQ* operon may play important roles in response to environmental changes. In our present study, S. mutans strains were cultured in an anaerobic environment, eliminating the interference of oxygen. When exposed to oxygen, the expression level of the *rcrRPQ* operon and the observed phenotype(s) of our strains, including biofilm formation and sugar utilization, might be different from those under anaerobic conditions. These speculations remain to be further verified in future studies.

The MarR family transcriptional regulators are widely distributed in bacteria. They regulate various functions, such as resistance to antibiotics and oxidative stressors, degradation of harmful chemicals, and the expression of virulence genes (23). These proteins mainly bind to recognizable palindromic sequences within the intergenic region of divergent genes. Binding leads to reduced gene expression, as the RNA polymerase is hindered from binding to the promoter (23). Previous studies have shown that RcrR, as one of the MarR family members, functions as a repressor to regulate *rcrP* and *rcrQ* expression in the *rcrRPQ* operon by binding to its promoter region in S. mutans (16, 24). In this study, the expression levels of the two PTS operons were downregulated when the *rcrR* gene was knocked out, suggesting that RcrR directly functioned as an activator to regulate the gene expression of the two PTS operons. The results of the EMSA (Fig. 9A) showed that RcrR was able to bind to promoter regions of the two PTS operons. Moreover, keeping in mind the results of qRT-PCR (Fig. 9B) *in vivo* (Fig. 9D), it is probable that RcrR functions as an activator binding to the promoter regions to recruit or stabilize RNA polymerase, or alter promoter conformation for activating transcription, via a mechanism similar to that of the CueR regulator (25). Nonetheless, the actual mechanism underlying activation after RcrR binding with the two promoter regions should be further explored.

Our DNase I footprinting assays results showed that there were three fragments (oligonucleotides 1, 21, and 22; 16 nt) protected by RcrR and that their sequences were highly identical, which further verified and supplemented the conserved DNA sequence (24). Meanwhile, oligonucleotide 21 plus oligonucleotide 22 was a palindromic sequence with high AT content, suggesting that RcrR prefers binding to DNA regions with high AT content. This preference was also similar to those of the MarR family regulators CouR (26) and FabT (27).

S. mutans appears to have developed diverse strategies for sugar utilization in response to complex environmental changes in the human oral cavity. A series of carbohydrate transporter systems have been found in S. mutans UA159, including 14 PTS systems and 4 sugar ABC transport systems (11, 28). The PTS system consists of three components, namely enzyme I (EI), EII (EIIA-D), and a histidine-containing phospho-carrier protein (HPr). Glucose binds to EII and is transported into the cell membrane, where HPr phosphorylates it to form glucose 6-phosphate (Glc-6P), which then immediately enters the glycolysis pathway (6, 29). The well-characterized mannitol operon contains *SMU_1185* (encoding a mannitol-specific EIIBC PTS component), *SMU_1184c* (a transcriptional regulator, antiterminator), *SMU.1183* (encoding a mannitol-specific EIIA PTS component), and *SMU_1182* (encoding a mannitol-1-phosphate dehydrogenase) (28). The trehalose-specific PTS system contains *SMU_2037* (encoding a trehalose-6-phosphate hydrolase), *SMU_2038* (encoding a trehalose-specific EIIABC PTS component), and *SMU_2040* (encoding a GntR family regulator, TreR) (13, 28). These components of the two operons play a critical role in sugar transportation. In this study, deletion of *rcrR* resulted in decreased expression of these components. Our data also showed that the deletion of binding sites could result in a low growth rate and low final yield of S. mutans when cultured in TV plus sugar medium (Fig. 10B, C, G, and H). Given that RcrR binds to the promoter regions of the two operons, it is easy to think that the deletion of binding sites abolished the activation ability of RcrR upon binding with the promoter regions, consequently leading to decreased expression of the two operons and a decreased ability to transport multiple sugars. In addition, we also speculate that the deletion of *rcrR* mainly influenced sugar uptake, resulting in a decrease in substrate sources, energy for bacterial growth, and sugar metabolism in S. mutans, as genes related to sugar metabolism showed no significant changes based on RNA-seq results. These results possibly explain the reason for the delay in bacterial growth and decreased biofilm formation ability. However, this hypothesis should be further tested.

Sugar transport and metabolism are regulated by different mechanisms (15). Organisms can express and coordinate the regulation of multiple sugar uptake systems in response to complex environments. Previous studies have postulated that PTS systems are widely regulated by various factors, such as FruR (9), StsR (12), TreR (13), CelR (14), and NigR (15). For example, deletion of *stsR* changes the expression levels of PTS and ABC transporter genes, including mannitol- and trehalose-specific PTS systems (12). Other studies have also reported that the mannitol- and trehalose-specific PTS systems are regulated by MtlR and TreR, respectively (13, 30). Here, RcrR, as an activator, also regulated these two operons. As such, these results suggest that RcrR, probably interacting with proteins (StsR or TreR), can coregulate the transcription of *SMU_2037* and *SMU_2038*.

In summary, S. mutans has multiple pathways to coordinate sugar transport and metabolism in response to complex environmental conditions. The findings demonstrate that RcrR is a transcription activator with pleiotropic effect on bacterial growth, biofilm formation, and multiple sugar transportation, which are all important virulence traits of S. mutans. Based on this, it is of paramount interest to determine the specific signals that can induce the RcrR-related regulatory pathway. Notably, this study also decrypts the physiological functions of RcrR as a potential target for controlling biofilm formation and multiple sugar uptake for the better prevention of dental caries.

## MATERIALS AND METHODS

### Bacterial strains and their growth conditions.

All bacterial strains and plasmids used in this study are listed in Table S1 in the supplemental material. S. mutans UA159 and its derivatives were routinely grown in BHI broth (BD, USA) or BHI agar at 37°C in an anaerobic incubator (80% N_2_, 10% H_2_, and 10% CO_2_). Escherichia coli strains were grown in Luria-Bertani medium at 37°C under aerobic conditions.

10.1128/mSystems.00788-21.2 TABLE S1 Strains, plasmids, and primers used in this study. Download Table S1, XLSX file, 0.01 MB. Copyright © 2021 Gong et al. 2021 Gong et al. https://creativecommons.org/licenses/by/4.0/ This content is distributed under the terms of the Creative Commons Attribution 4.0 International license.

### Construction of in-frame mutants.

An in-frame *rcrR* deletion (Δ*rcrR*) mutant strain was constructed using S. mutans UA159. Construction was performed via a two-step transformation procedure (5, 20). PCR and DNA sequencing methods were used to confirm whether a correct *rcrR* deletion mutant strain was obtained. Mutants of Δ*1185p* and Δ*2038p* were obtained following the same method. All primers used here are listed in Table S1.

### Construction of *rcrR* complement and overexpression strains.

The *rcrR* coding sequence, including its promoter region (upstream 490 bp) was cloned into a SacI/SalI-digested E. coli-Streptococcus shuttle vector, pDL278 (31). The recombinant plasmid *pDL278-rcrR* was then transformed into an *rcrR* mutant strain to generate the complement strain Δ*rcrR*/*pDL278-rcrR*. The transformation efficiency (or genetic competence) is reduced because of the deletion of *rcrR* (16); therefore, we constructed the complement strain (Δ*rcrR*/*pDL278*-*rcrR*) using electroporation. *pDL278-rcrR* was also transformed into UA159 to generate the overexpression strain UA159/*pDL278-rcrR*, as previously described (5). PCR and DNA sequencing were performed to confirm whether the correct strains were obtained. The primers used in this study are listed in Table S1.

### Growth curves.

Cultures grown overnight in BHI medium were diluted to 1:10 in fresh BHI and grown to the mid-exponential phase (optical density at 600 nm [OD_600_] ≈ 0.5). They were then diluted to 1:100 into fresh BHI, 1/2 BHI, and 1/4 BHI media. Cell growth was monitored using Multiskan GO (Thermo Fisher Scientific, USA) for 12 h under anaerobic conditions (80% N_2_, 10% H_2_, and 10% CO_2_). OD_600_ was measured at 1-h intervals. The growth of the strains was further assessed in the presence of different carbohydrates. Cultures grown overnight in BHI medium were diluted to 1:10 in fresh BHI and grown to the mid-exponential phase (OD_600_ ≈ 0.5). They were then diluted to 1:100 in a tryptone-vitamin (TV) base medium supplemented with 1% (wt/vol) glucose, galactose, lactose, fructose, maltose, or trehalose (17). The base TV medium contained 3.5 g of tryptone/100 ml, 0.04 μg of *p*-aminobenzoic acid/ml, 0.2 μg of thiamine-HCl/ml, 1 μg of nicotinamide/ml, and 0.2 μg of riboflavin/ml. Cell growth was monitored using Multiskan GO for 24 h under anaerobic conditions. OD_600_ was then measured at 2-h intervals. Overnight cultures of strains were diluted to 1:10 in fresh BHI and grown to the mid-exponential phase (OD_600_ ≈ 0.5); subsequently, the cultures were diluted to 1:10 and transferred to fresh TV medium supplemented with glucose, galactose, lactose, fructose, maltose, mannitol, or trehalose (1%, wt/vol) (Fig. 10). Cell growth was monitored using Multiskan GO for 12 h under anaerobic conditions. OD_600_ was measured at 2-h intervals. Each analysis was performed in triplicate, and the representative growth curves were plotted.

### Biofilm detection by crystal violet staining assay.

As described previously (32), cultures grown overnight were diluted to 1:10 in fresh BHI and grown to an OD_600_ of approximately 0.5 to 0.6. The log-phase cells were then diluted to 1:100 in fresh BHI plus 1% sucrose (BHIS; wt/vol) or TV plus 1% sucrose (TVS) and cultured in sterile 48-well microtiter plates for 24 h. The cells were then dumped out after incubation by turning the plate over, followed by gently submerging the plate in a small tub of water. The water was then drained out, and 0.01% crystal violet was added to stain the biofilm for 15 min. The plate was then rinsed 3 or 4 times by submerging in water. Ethanoic acid (30%) was then added to solubilize the dye for 15 min. Finally, 100 μl of ethanoic acid solution was transferred to a new plate, and its absorbance at 570 nm was measured.

### Confocal laser scanning microscopy analysis of biofilm.

Biofilms were grown on a glass-bottomed petri dish (NEST, China) in BHIS or TVS medium for 24 h. Bacterial cells and EPS from biofilms were then labeled with 5 μM SYTO 9 and 1 μM Alexa Fluor 647 (Invitrogen, USA), respectively (33). Alexa Fluor 647-labeled dextran conjugate was then added to the culture medium before inoculation. The medium was subsequently decanted after incubation, and the petri dishes were washed twice with 0.85% NaCl. The bacteria were then stained with 50 μl SYTO 9 for 15 min, and then the residual dye was washed twice with 0.85% NaCl. Confocal imaging was captured using an FV3000 instrument (Olympus, Japan) with a 60× oil immersion objective. The image collection gates were set at 650 to 750 nm for Alexa Fluor 647 and 500 to 540 nm for SYTO 9. During imaging, Gain (1.0×), HV (365 V for SYTO 9; 410 V for Alexa Fluor 647), and offset (4%) were kept constant. Z sections were used to determine the biofilm thickness. Three-dimensional reconstruction of biofilms and calculation of bacteria and EPS biomass were performed using Imaris 9.0.1 and ImageJ contained in Comstat 2.1, respectively (34).

### Transcriptome sequencing and data analysis.

For transcriptome analysis, the S. mutans UA159 and S. mutans Δ*rcrR* strains were routinely grown at 37°C under anaerobic conditions in BHI medium until an OD_600_ of 0.5 was reached. They were then centrifuged (4,000 × *g*, 4°C, 10 min) and snap-frozen in liquid nitrogen for 15 min. They were subsequently submitted to Majorbio Co., Ltd. (Majorbio, China) for genome-wide RNA sequencing. Three independent total RNA extracts of each sample were extracted using TRIzol reagent according the manufacturer’s instructions (Invitrogen, USA), and genomic DNA was removed using DNase I (TaKaRa, Japan). Then, RNA quality was determined using a 2100 Bioanalyzer (Agilent, USA) and quantified using an ND-2000 instrument (NanoDrop Technologies, USA). cDNA libraries were constructed from enriched mRNA samples using the TruSeq RNA sample prep kit (Illumina, USA). rRNA depletion from total RNA was performed using the Ribo-Zero magnetic kit (Epicentre, USA), and the mRNA was chemically fragmented to short pieces (200 nt) using a 1× fragmentation solution (Ambion, USA) for 2.5 min at 94°C. Double-stranded cDNA was produced using the SuperScript double-stranded cDNA synthesis kit (Invitrogen, USA) with random hexamer primers (Illumina, USA). Then, the synthesized cDNA was subjected to end repair, phosphorylation, and “A” base addition according to Illumina’s library construction protocol. Libraries were selected for cDNA target fragments of 200 bp on 2% low-range Ultra agarose followed by PCR amplification using Phusion DNA polymerase (NEB, USA) for 15 PCR cycles. After quantification by TBS380, the paired-end RNA-seq sequencing library was sequenced with the Illumina HiSeq X Ten instrument (2 × 150-bp read length). Genes with a fold change of >2.0 and a *P* value of <0.05 were selected for further gene expression pattern discovery. All analyses were performed using the free online Majorbio cloud platform (www.majorbio.com). The DEGs were further used for GO enrichment analysis of functional classifications and KEGG pathway analysis.

### Expression and purification of the recombinant RcrR protein.

The *rcrR* gene was cloned into pET28a to produce recombinant vectors (pET28a-*rcrR*). The EcoRI and XbaI restriction sites were used. The RcrR protein was then purified using the His tag protein purification kit (Beyotime, China), following the manufacturer’s protocol. The concentration of purified His-RcrR protein was determined by measuring its spectrophotometric absorbance at 280 nm. The primers used are listed in Table S1.

### DNA substrate preparation and EMSA.

The *rcrR* promoter (551 bp) was divided into three DNA fragments, P1 (184 bp), P2 (184 bp), and P3 (183 bp). P1, P2, P3, the mannitol-specific promoter (184 bp), and the trehalose-specific promoter (173 bp) were acquired by PCR. The DNA fragments (20 pmol) were then incubated with various amounts of RcrR proteins in a total volume of 20 μl EMSA buffer for 30 min on ice. The buffer contained 20 mM Tris-HCl (pH 7.0), 100 mM NaCl, and 5% (vol/vol) glycerol. The DNA-protein complexes were then resolved by electrophoresis on a 5.8% (wt/vol) nondenaturing polyacrylamide gel in 0.5× Tris-borate-EDTA (TBE) buffer at 110 V for 60 min. The gels were dyed with ethidium bromide for 20 min, and images were captured using a ChemiDoc touch imaging system (Bio-Rad, USA). The primers used in the study are listed in Table S1.

### DNase I footprinting assays.

The P3 fragment was amplified using 6-carboxyfluorescein (FAM)-labeled primers. Amplified fragments were then subjected to a binding reaction similar to that in EMSA. DNase I footprinting was then performed (35). The reaction mixtures, composed of 20 pmol of gene products and either 0, 80, or 160 pmol of RcrR, were treated with DNase I (0.015 U) at 37°C for 5 min. The samples were then extracted with phenol, precipitated with ethanol, and eluted in 15 μl of distilled water. The fragments were analyzed using a 3730XL DNA analyzer (Applied Biosystems, Tsingke, China). The two operon promoters were used to search for a generally conserved motif for RcrR binding using MEME Suite 5.1.1.

### RNA extraction and quantitative reverse transcription-PCR.

Bacterial strains were grown overnight in BHI broth and diluted to 1:10 in fresh BHI. They were allowed to grow to an OD_600_ of 0.5 and then harvested for total RNA isolation. Extraction was performed using the MasterPure complete DNA and RNA purification kit (Lucigen, USA) following the manufacturer’s protocol. cDNA was synthesized using the PrimeScript RT reagent kit with the gDNA Eraser kit (TaKaRa, Japan) according to the manufacturer’s instructions. qRT-PCR was performed on a QuantStudio 6 Flex real-time PCR system (ABI, USA) using TB Green Premix Ex Taq II (TaKaRa, Japan). The QuantStudio 6 Flex software determined threshold cycles and dissociation curves. The determination was conducted to ensure that only one PCR product was amplified and detected. The differential expression fold was quantified following the comparative threshold cycle (2^−ΔΔ^*^CT^*) method with 16S rRNA as an internal standard. The primers used in this study are listed in Table S1.

### Statistical analysis.

All experiments were performed in triplicate and separately reproduced three times. Data were analyzed using SPSS statistical software (version 20 for Windows; SPSS, Inc., Chicago, IL). Student’s *t* test was used to compare data between two groups. Analysis of variance (ANOVA) and Tukey’s test were performed to compare data between multiple groups. A *P* value of <0.05 indicated that there were significant differences between groups.

### Data availability.

The raw data from the transcriptomic sequencing analysis of S. mutans UA159 and the S. mutans Δ*rcrR* mutant have been deposited in the NCBI Sequence Read Archive (SRA) database under BioProject accession number PRJNA749547.

10.1128/mSystems.00788-21.8 TABLE S7 GO enrichment analysis of the upregulated genes in the S. mutans Δ*rcrR* strain (*P < *0.05). Download Table S7, XLSX file, 0.01 MB. Copyright © 2021 Gong et al. 2021 Gong et al. https://creativecommons.org/licenses/by/4.0/ This content is distributed under the terms of the Creative Commons Attribution 4.0 International license.
