# Supplementary figures and images for: Transcriptional Profiling Reveals the Importance of RcrR in the Regulation of Multiple Sugar Transportation and Biofilm Formation in Streptococcus mutans
Source: mSystems. 2021 Aug 24;6(4):e00788-21. doi: 10.1128/mSystems.00788-21 (PMC8407328; doi:10.1128/mSystems.00788-21)

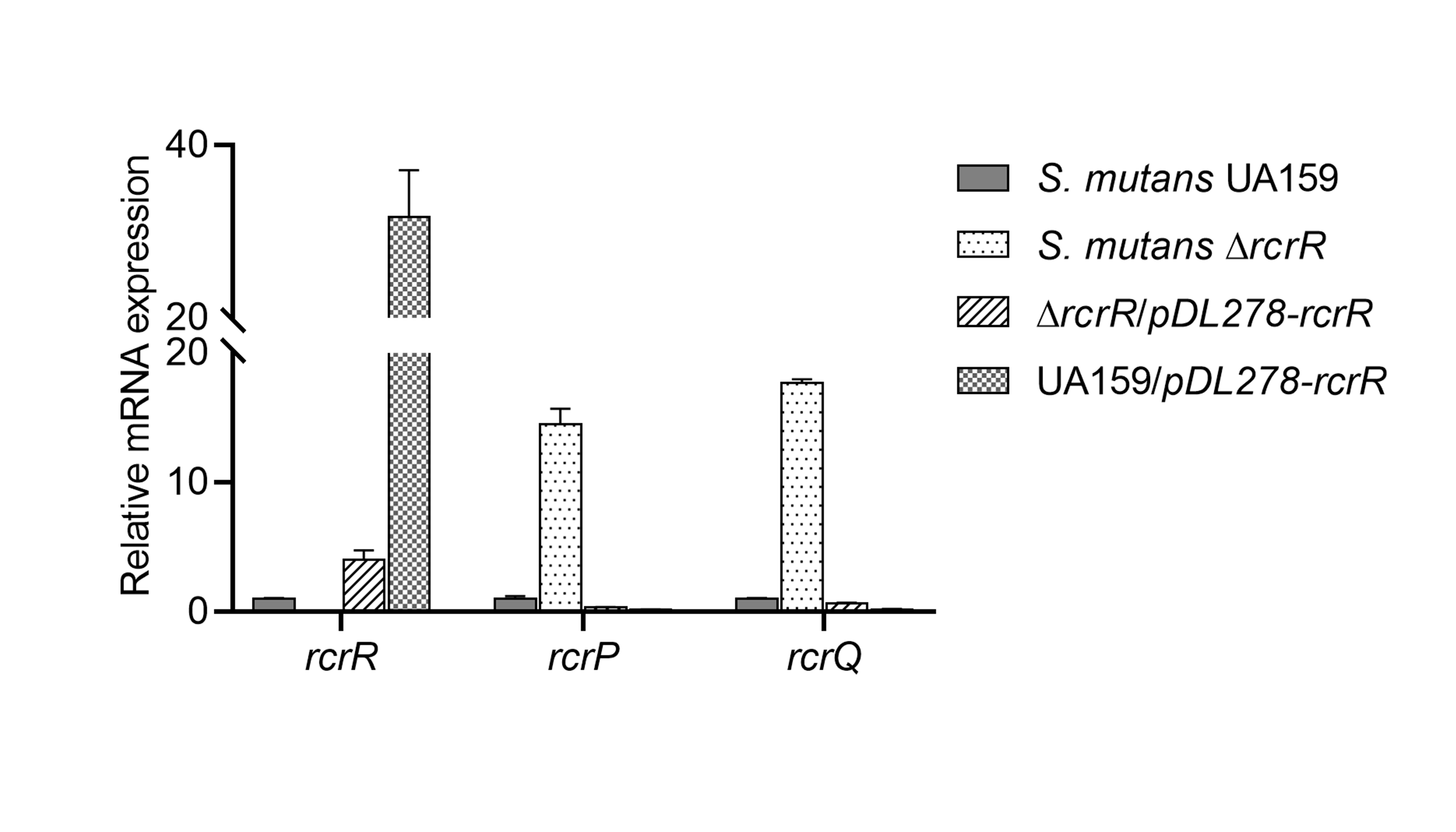

Supplement: FIG S1 [file msystems.00788-21-sf001.tif]
